# Lingering Dynamics of Type 2 Diabetes Mellitus Red Blood Cells in Retinal Arteriolar Bifurcations

**DOI:** 10.3390/jfb13040205

**Published:** 2022-10-27

**Authors:** Lili Long, Huimin Chen, Ying He, Lizhong Mu, Yong Luan

**Affiliations:** 1 Key Laboratory of Ocean Energy Utilization and Energy Conservation of Ministry of Education, School of Energy and Power Engineering, Dalian University of Technology, Dalian 116000, China; 2 Department of Anesthesiology, The First Affiliated Hospital of Dalian Medical University, Dalian 116011, China

**Keywords:** diabetes mellitus erythrocyte, retinal vessel, fluid–structure interaction, lingering time

## Abstract

It has been proven that the deformability of red blood cells (RBC) is reduced owing to changes in mechanical properties, such as diabetes mellitus and hypertension. To probe the effects of RBC morphological and physical parameters on the flow field in bifurcated arterioles, three types of RBC models with various degrees of biconcave shapes were built based on the in vitro experimental data. The dynamic behaviors of the RBCs in shear flow were simulated to validate the feasibility of the finite element-Arbitrary Lagrangian–Eulerian method with a moving mesh. The influences of the shear rate and viscosity ratios on RBC motions were investigated. The motion of RBCs in arteriolar bifurcations was further simulated. Abnormal variations in the morphological and physical parameters of RBCs may lead to diminished tank-tread motion and enhanced tumbling motion in shear flow. Moreover, abnormal RBC variations can result in slower RBC motion at the bifurcation with a longer transmit time and greater flow resistance, which may further cause inadequate local oxygen supply. These findings would provide useful insights into the microvascular complications in diabetes mellitus.

## 1. Introduction

Diseases related to microcirculation, such as diabetes mellitus (DM), hypertension, and atherosclerosis, are major health problems in modern society. Retinopathy is one of the most common complications of diabetes mellitus (DM). Retinal vasculature is readily displayed in vivo and can be directly observed and characterized by non-invasive means. Health assessment of the retinal vasculature is a potential biomarker of pathological processes in the eyes and other organs [1].

Microvasculature is one of the basic components of microcirculation, and its size is comparable to that of a red blood cell (RBC). RBCs are the main suspension component of blood, functioning as transporters of oxygen and carbon dioxide between the lungs and peripheral tissues. RBCs are composed of a viscous fluid and an elastic membrane encapsulating the viscous fluid (hemoglobin solution). The RBC membrane is linked by a combination of a phospholipid bilayer and a cytoskeleton (spectrin network) [2,3], with the composite properties of a phospholipid bilayer and a protein network determining the RBC biconcave shape, resulting in membrane elasticity and biomechanical properties [4] as well as controlling RBC deformability [5]. Normal RBC deformability is a key determinant of proper blood flow and function in microcirculation [6].

Several medical studies have demonstrated that, compared to healthy RBCs, the concave depth, diameter, and deformation index of RBCs in DM decreased, while the stiffness and viscosity increased [7]. Ciasca et al. [8] measured the viscoelasticity of RBCs on a nanoscale and found increased viscosity and stiffness of diabetic RBC membranes compared to healthy RBCs. Jin et al. [9] and AlSahi et al. [10] studied the effect of DM on RBC morphology by applying atomic force microscopy and fluorescence spectroscopy, and pointed out that excessive glucose in the blood would lead to a swollen RBC shape. In addition, excessive glucose causes RBCs aggregation, which eventually leads to increased viscosity and slow the mobility of RBCs. Consequently, examining the effects of RBC morphological and physical parameters on the flow field may enhance the basic understanding of RBC behavior under healthy and DM conditions.

Fluid–structure interaction (FSI) is the most ideal method for studying the mechanism of RBC motion in addition to in vitro experiments. Pozrikidis et al. [11] in 1995 developed a simulation of capsule deformation in shear flow using the boundary integral method. Wei et al. [12] applied the immersed boundary-lattice Boltzmann method to study RBC motion and deformation in two-dimensional capillaries and calculated NO transport properties across the RBC membrane. In a recent study, Balogh et al. [13] employed the immersed-boundary method to present multiple RBCs motions in near-physiological three-dimensional microvascular networks. Most studies on RBCs have ignored the thickness of RBC membranes and treated RBCs as elastic capsules wrapped in a thin shell with no thickness. Abnormal changes in the morphology and mechanical properties of RBC can adversely affect RBC deformation.

Therefore, we considered the RBC membrane thickness and used classical shear flow to validate the feasibility of the finite element-Arbitrary Lagrangian–Eulerian (ALE) algorithm for calculating the RBC motion. Subsequently, we calculated the motion of single and multiple RBCs in retinal bifurcating arterioles. Meanwhile, the pressure drop at the bifurcation and the lingering time of the RBC at the bifurcation were quantified and analyzed.

## 2. Materials and Methods

### 2.1. Retinal Vessel Model

The circulatory system plays a principal role in transporting blood, which contains oxygen and nutrients indispensable for the growth and maintenance of the body, to the immediate vicinity of the tissues of the organs [14]. The branching vascular parameters of retinal arterioles can be described by the power law:

(1) $$d_{0}^{k}=d_{1}^{k}+d_{2}^{k}$$

where k is the vessel index, $d_{0}$ is the parent vessel diameter, and $d_{1},d_{2}$ are the child branch vessel diameters, respectively. The branching power law was derived by Murray and Zamir according to the principle of minimal work of the arterial system [15,16]. Several studies have reported k values varying between 2 and 3 [17,18,19]. Here, the k value was taken as 2.1 [20].

The asymmetry index is used to describe the relationship between the child vessels:

(2) $$\psi =d_{2}/d_{1}$$

Diameter ratio:

(3) $$\psi_{1}=d_{1}/d_{0}$$

(4) $$\psi_{2}=d_{2}/d_{0}$$

Bifurcation angle in accordance with the minimum pumping power and volume:

(5) $$\cos\theta_{1}=\frac{\psi_{1}^{-4}+1+\psi^{4}}{2\psi_{1}^{-2}}$$

(6) $$\cos\theta_{2}=\frac{\psi_{1}^{-4}+\psi^{4}-1}{2\psi^{2}\psi_{1}^{-2}}$$

Length of the retinal vessels:

(7) $$l=7.4{\left(d_{i}/2\right)}^{1.15},i=0,1,2$$

The diameter, length, and bifurcation angle of each segment of the child vessel can be calculated, given the diameter and asymmetry ratio of the parent vessel. In this study, the parent vessel diameter $d_{0}$ is set to 9.5 μm [21], and the asymmetry index ψ is 1, which means that the child vessels are perfectly symmetrical.

### 2.2. RBC Model

An RBC is considered a viscous fluid wrapped by a viscoelastic membrane with a biconcave shape. The model equation is as follows [22]:

(8) $${\left(x^{2}+y^{2}+a^{2}\right)}^{2}-4a^{2}x^{2}=b^{4}$$

where a and b are geometric parameters related to the RBC diameter d and height h [23]:

(9) $$a^{2}=d^{2}-h^{2}/8,b^{2}=d^{2}+h^{2}/8$$

Here, the RBC membrane thickness was quoted from in vitro experimental studies, setting as 54 nm [24,25]. We define three types of RBCs with different degrees of biconcave shapes based on in vitro experimental studies: the healthy red blood cell (H-RBC), pre-diabetic red blood cell (P-RBC), and diabetic red blood cell (D-RBC), whereas for a detailed description of the RBC model parameters, we refer to [26].

### 2.3. Governing Equations

Blood is an incompressible fluid, and its flow equation is described by the Navier–Stokes equation:

(10) $$\rho_{fluid}\left[\frac{\partial u_{fluid}}{\partial t}+\left(u_{fluid}\times \nabla \right)u_{fluid}\right]=\nabla \times \left[-pI+\mu (\nabla u_{fluid}+{\left(\nabla u_{fluid}\right)}^{T})\right]$$

(11) $$\nabla \times u_{fluid}=0$$

where $\rho_{fluid}$ is the plasma density, $u_{fluid}$ is the plasma velocity vector, p is pressure, and μ is viscosity. Plasma density and cytoplasmic density were chosen to have the same magnitude, 1060 kg/m^3^. The viscosity ratio λ of cytoplasm viscosity and plasma viscosity was defined as $\lambda ={\mu_{cytoplasm}/\mu }_{plasma}$, and the physiological viscosity ratio $\lambda_{c}$ was 5 [27].

The RBC membrane is isotropic and viscous, and the behavior of the RBC fluid interaction can be described by the elastic dynamic equation:

(12) $$\rho_{solid}\frac{\partial^{2}U_{solid}}{\partial t^{2}}=\nabla \sigma +F_{V}$$

where $\rho_{solid}$ is the RBC membrane density, $U_{solid}$ is the RBC membrane displacement vector, σ is the stress tensor of the RBC membrane, and $F_{V}$ is the force per unit volume of RBCs. The stress–strain relationship is expressed as follows:

(13) $$\sigma =G\varepsilon +\eta \frac{d\varepsilon }{dt}$$

where G represents the RBC membrane shear modulus and G=E/(2(1+ν)). Notably, E and ν are Young’s modulus and Poisson’s ratio of the RBC membrane, respectively. η is the viscosity of the RBC membranes. ε is the strain tensor of the RBC membrane, and its equation is as follows:

(14) $$\varepsilon (t)=\frac{\sigma_{0}}{G}(1-e^{-t/\tau })$$

where $\sigma_{0}$ denotes the initial strain tensor of the RBC membrane. τ is the relaxation time of the RBC membrane, τ=η/G.

The interfaces between the RBC membrane, plasma, and cytoplasm are FSI boundaries. Two-way coupling is captured along the FSI boundary by the fluid applying forces on the RBC membrane, and the RBC membrane displacement imposes a moving wall boundary condition on the fluid. These conditions can be expressed as:

(15) σ×n=Γ×n

(16) $$u_{fluid}=u_{soild}$$

(17) $$u_{soild}=\frac{\partial U_{solid}}{\partial t}$$

where Γ is the force of the fluid acting on the RBC membrane, $\Gamma =\left[-pI+\mu (\nabla u_{fluid}+{\left(\nabla u_{fluid}\right)}^{T})\right]$, n is the normal vector at the fluid–structure boundary, and $u_{solid}$ refers to the velocity vector of the RBC membrane.

The Neo-Hookean model of hyper-elastic material was chosen for the RBC membrane [28,29], and the Kelvin–Voigt model was adopted to realize the viscoelasticity of the RBC membrane. The RBC membrane density is set to 1090 kg/m^3^ [30]. The parameters used in the model are shown in Table 1.

### 2.4. Boundary Conditions

**Figure 1:**
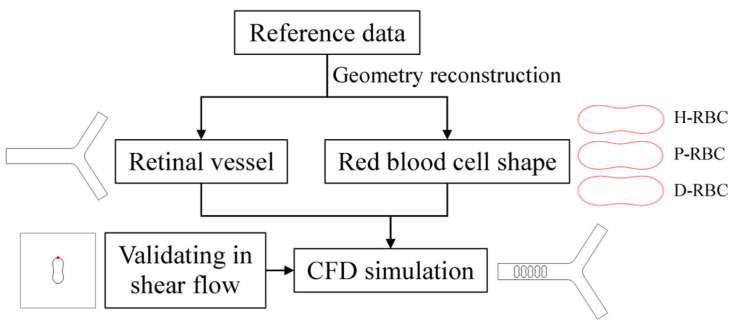
Numerical simulation framework.

The numerical simulations were carried out by the commercial software COMSOL Multiphysics 5.6 on AMD EPYC 7452 64-Core @2.35GHz computer node processor. COMSOL Multiphysics is a largescale finite element analysis software for multi-physics simulation, which is widely used in various fields of scientific research and engineering project calculation. A fully developed velocity profile was set at the inlet, and the mean velocity was set to 0.26 cm/s, which refers to a similar vessel size [21,31]. The outlet pressure was set to 0 Pa. A moving mesh model was used to track the fluid deformation. Meshes are performed using an ALE mesh, where the Eulerian mesh traces the fluid, and the Lagrangian mesh describes the RBC membrane. RBCs undergo large deformations during movement, resulting in mesh distortion. Therefore, an automatic remeshing technology was adopted in the calculation when the mesh quality fells below a specified value of 0.2, and the calculation continued after the solver remeshed. The meshes of the RBC membrane were particularly refined. The numerical results are considered mesh-independent when the difference in the pressure drop between two consecutive simulations is less than 2%. Figure 1 shows the flow chart of numerical simulation framework in this study.

The values of RBC morphology and blood properties used were taken from the published literature, as summarized in Table 2.

## 3. Results

In the present study, we simulated the dynamic behavior of H-RBC, P-RBC, and D-RBC in shear flow based on a finite element ALE method with a moving mesh, to validate the feasibility of calculating RBC motion and explore the influence of shear rate υ and viscosity ratios λ on RBC motion. Subsequently, we simulated RBC motion in a retinal bifurcating arteriole to examine the effects of RBC morphology and physical parameters on the hemodynamics of the bifurcating vessels.

### 3.1. Dynamic Behavior of RBC in Shear Flow

**Figure 2:**
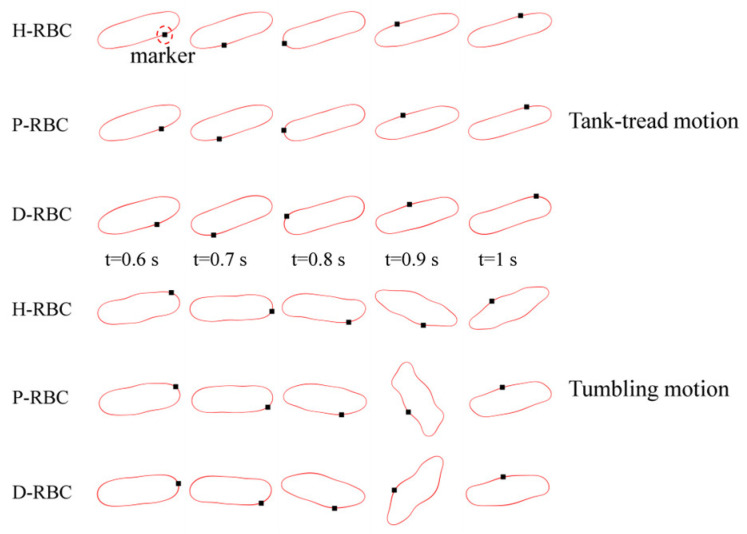
Tank-tread (TT) motion (**top**) and tumbling (TB) motion (**bottom**).

RBC in shear flow has identified two major dynamic behaviors: tank-tread (TT) motion and tumbling (TB) motion (Figure 2). It can be observed that the TT motion of the marker point and TB motion in D-RBC are the fastest. In this study, we focused on the form of RBC motion at different viscosity ratios and different shear rates and compared the differences among the three groups of RBC motion. Based on the previous studies, we simulated RBC motion in shear flow at shear rates in υ=50,100,150 1/s corresponding to viscosity ratios λ=1,3,5,7,9 [36,37]. The shear flow region was 20 μm × 20 μm, and the RBC was located at the center. The upper and lower walls impose opposite velocities but the same magnitude to form the shear flow, and the left and right sides are period boundaries.

**Figure 3:**
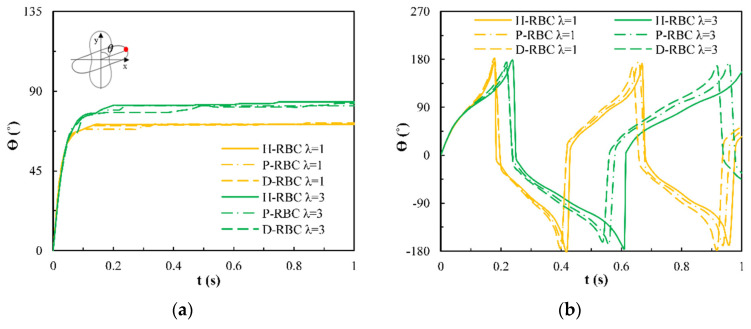

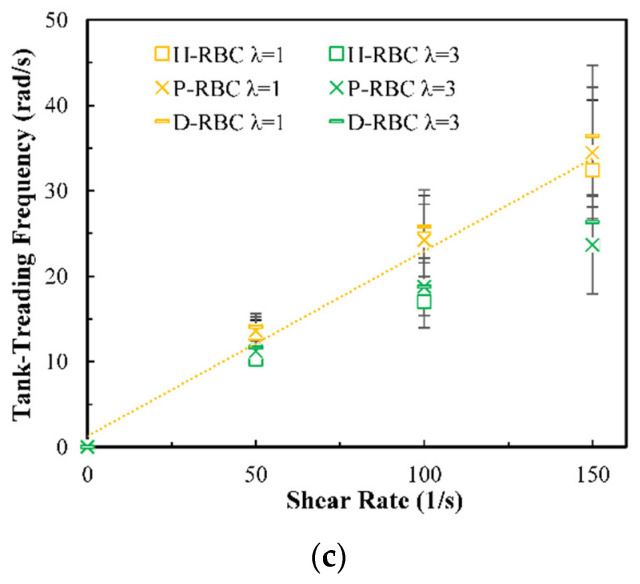
TT motion for λ=1,3 at υ=50 1/s. (**a**) Inclination angle in shear flow λ=1,3 of RBC; (**b**) trajectories of the marked point on the RBC membrane over time; (**c**) functional dependence of RBC TT frequency with respect to υ.

Under the shear force, when $\lambda ≺\lambda_{c}$, the RBC begins to rotate, after rotating to a certain inclination angle, it maintains a stable state. The RBC membrane undergoes TT motion. The concave point of RBC gradually disappears, and its shape changes irregularly, Subsequently, it stretches along the inclination direction [38]. Figure 3a provides the inclination angle of three groups of RBCs in shear flow when λ=1,3 at υ=50 1/s. When $\lambda \geq \lambda_{c}$, the RBC only undergoes TT motion in shear flow. Notably, with λ increasing slowly, the inclination angle increases [39,40,41]. This agrees with the previous studies [39] that the form of RBC motion is highly dependent on λ. Figure 3b shows the angle trajectory of the marker point on RBC membrane over time at υ=50 1/s. Notably, it can be found that lower λ has a short period, D-RBC moves a little faster than P-RBC and H-RBC. Figure 3c shows TT frequency of three groups of RBCs increases linearly with increasing υ [35]. The slope of D-RBC is the largest, followed by P-RBC.

**Figure 4:**
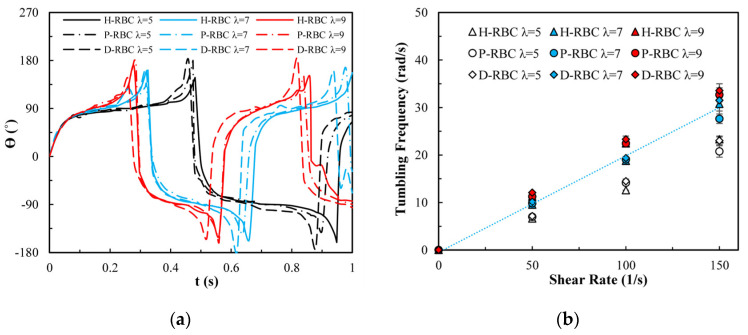
TB motion for λ=5,7,9 at υ=50 1/s. (**a**) TB angle of RBC at λ=5,7,9 over time; (**b**) functional dependence of TB frequency with respect to υ.

Figure 4a shows the variation in the TB angle with time at υ=50 1/s. During $\lambda \geq \lambda_{c}$, the RBC mainly undergo TB motion. The rotation speed of the D-RBC was greater than that of the P-RBC and H-RBC. Higher λ has a faster TB speed and shorter TB period for all RBCs. We counted TB frequency at υ=50,100,150 1/s, as shown in Figure 4b. The TB frequency increased linearly with increasing υ. In contrast to TT motion, greater λ has a greater TB frequency. The TB frequency in D-RBC was higher than that in H-RBC and P-RBC. It can be clearly seen that the TB frequency of the D-RBC is the largest at any λ value. This is expected because the RBC shape and physical parameters have changed significantly, which makes a big difference to RBC motion.

### 3.2. Dynamics of an RBC in Flowing through a Retinal Bifurcating Vessel

**Figure 5:**
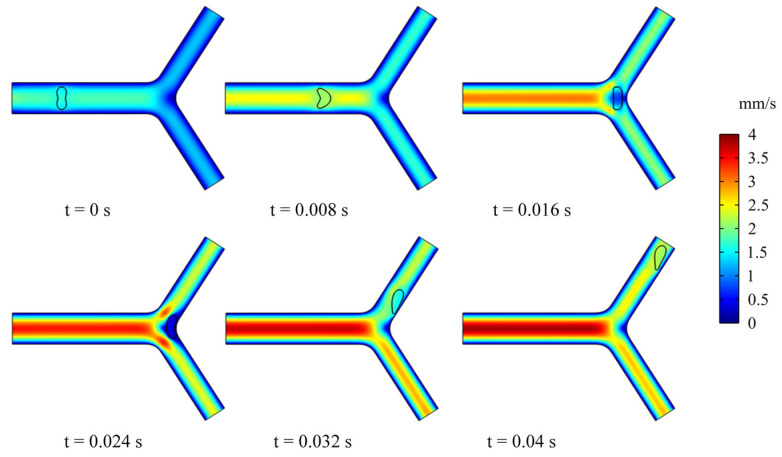
Snapshots of an RBC motion and deformation at different moments in the retinal bifurcating vessel.

In this section, we studied the dynamics and rheology of three groups of RBCs through the retinal bifurcating vessels at $\lambda =\lambda_{c}=5$, as shown in Figure 5. The RBC is released along the center of the vessel and gradually adopts the shape of a parachute in the parent vessel. After flowing into a child vessel with a diameter smaller than that of RBC, the RBC stretches to flow in an elongated bullet shape. As the RBC approaches the bifurcation, it immediately folds in the opposite direction, and we have observed that the RBC lingers for some time at the bifurcation.

**Figure 6:**
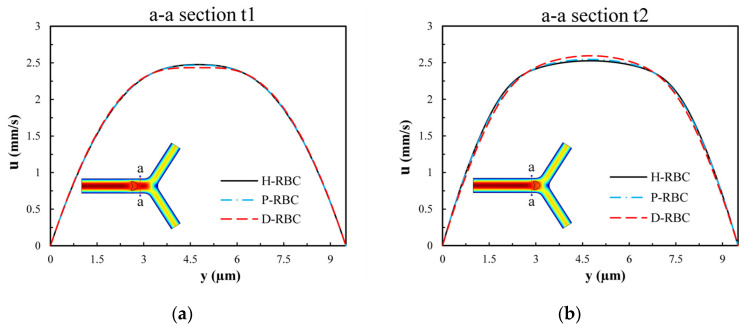
The velocity profile distribution of the a-a section: (**a**) when the RBC does not pass through the a-a section but close to it at the moment, t1; (**b**) when the RBC passes through the a-a section at the moment, t2.

To examine the influence of RBCs on the flow field, we extracted the velocity distributions at moments t1 and t2 of the a-a section near the bifurcation of the vessel. Figure 6 presents the velocity profile distribution of the a-a section at t1 and t2 moments (t1 < t2). The velocity profile in D-RBC is blunt compared to the other groups at the t1 moment shown in Figure 6a, while after RBC has passed through the a-a section, the velocity profiles in H-RBC and P-RBC are blunt compared to those in D-RBC, as shown in Figure 6b. This indicates that the RBC shape and physical parameters have little effect on the flow field during a single RBC moving. However, the redistribution of RBC may affect the flow field after approaching the bifurcation.

**Figure 7:**
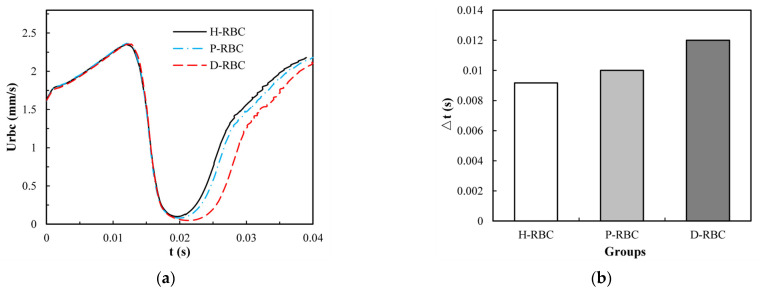
(**a**) The RBC velocity distribution in the retinal bifurcating vessel; (**b**) RBC lingering time in the retinal bifurcating vessel.

To take lingering into account, we refer to the definition of the lingering phenomenon of RBCs at the bifurcation in reference [42], indicating that the RBC lingers at the bifurcation if RBC velocity decreases severely near the bifurcation. The velocity of the three groups of RBCs increased slowly in the parent vessel, and close to the bifurcation, the RBC velocity decreased rapidly for some time, as shown in Figure 7a. The D-RBC velocity decreased more at the bifurcation as well as at the child vessel. Therefore, we withdrew the periods when the RBC velocity was less than 30 μm/s to quantify the lingering time of RBCs in each group, as shown in Figure 7b. The P-RBC lingering time was slightly longer than H-RBC, and the D-RBC lingering time was the longest. Thus, we predict that the biconcave shape of RBC decreases and the viscosity and stiffness of RBC increases, which may increase the resistance of vessel.

**Figure 8:**
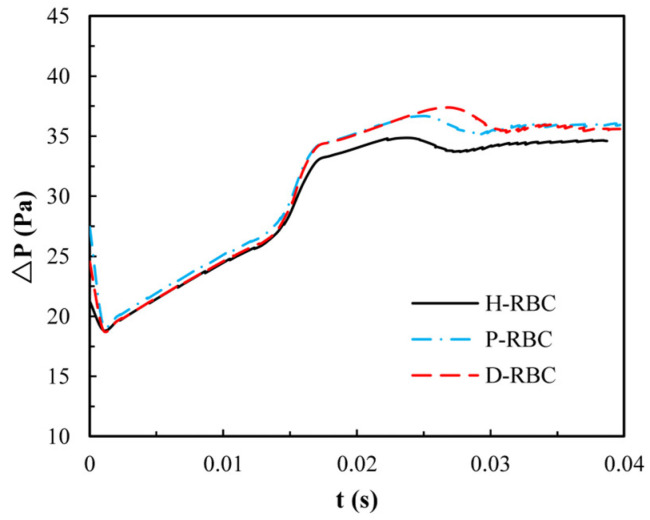
The pressure drop in the retinal bifurcating vessel.

Due to the lingering of RBCs at the bifurcation, we quantify the pressure drop changes during an RBC moving in the retinal bifurcating vessel over time, as shown in Figure 8. The pressure drop of three groups of RBCs is basically the same when the RBC moves in the parent vessel, whereas the pressure drop increases sharply when the RBC closes the bifurcation. Consequently, the pressure drop remains slightly increasing for a period of time at the bifurcation, when the RBC flows out of the bifurcation, and the pressure drop decreases slowly and increases slightly in the child vessel. The pressure drop in the D-RBC is greater than that in the H-RBC and P-RBC during RBC motion, which means that the D-RBC leads to a higher resistance at the bifurcation during motion.

### 3.3. Dynamics of Multi-RBCs in Flowing through a Retinal Bifurcating Vessel

**Figure 9:**
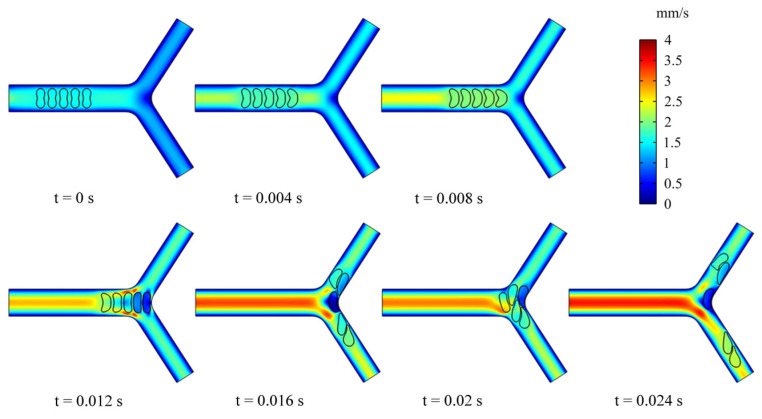
Snapshots of multi-RBCs motion and deformation at different moments in the retinal bifurcating vessel.

To examine the effect of multiple RBCs motions on the flow field, we simulated the dynamic behavior of five RBCs in the retinal bifurcating vessel at $\lambda =\lambda_{c}=5$. All RBCs were placed in the center of the parent vessel at an intercellular distance of 4 μm, as shown in Figure 9. When the RBCs reached the bifurcation, the preceding RBC was observed to undergo deformation. It is worth noting that the deformation of the preceding RBC affects the deformation of the following RBC, and the preceding RBC stays at the bifurcation for a long time, which can cause the next RBC to stack at the bifurcation and block the entrance of a child vessel. The deflection of the mass center determines which child branches the next RBC enters.

**Figure 10:**
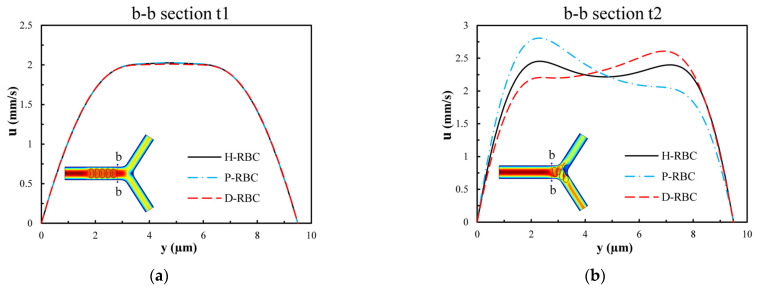
The velocity distribution of the b-b section: (**a**) when RBCs do not pass through the b-b section but close to it at the t1 moment; (**b**) when RBCs have just passed through the b-b section at the t2 moment.

Figure 10 presents the velocity distribution of the b-b section at t1 and t2 moments (t1 < t2). The velocity profile is blunted owing to the approach of the RBCs at the t1 moment in Figure 10a, while there is essentially no difference in the velocity distribution among the three groups of RBCs. Figure 10b shows the velocity distributions of section b-b when all RBCs have just passed through the b-b section at the moment, t2. For the H-RBCs, the velocity profile has two peaks, which are indicative of a relatively equal distribution of H-RBCs at the bifurcation. We predicted that this would result in a more homogenous distribution of the RBC flux in the child vessel. Conversely, the P-RBCs and D-RBCs have enhanced aggregation at the bifurcation, causing the velocity profile to be inclined toward the wall. This indicates that there is a more uneven RBC distribution in DM and hyperglycemia at the bifurcation, which may cause unequal redistribution of RBCs in the child vessel, resulting in large heterogeneity in the vessel hematocrit and a reduction in the average capillary discharge hematocrit.

**Figure 11:**
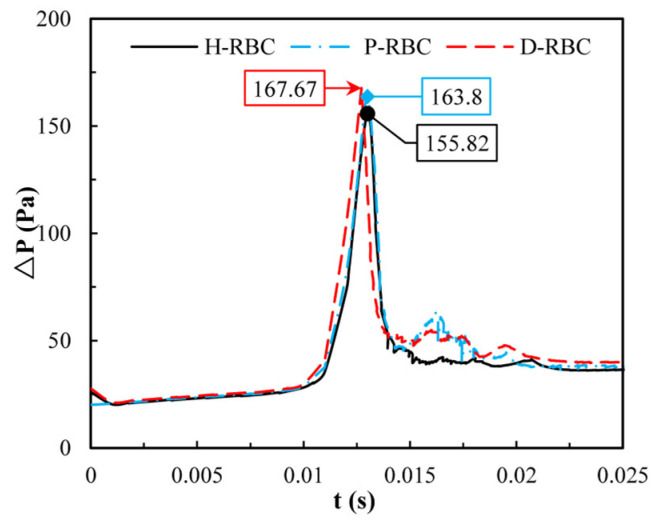
The pressure drop in the retinal bifurcating vessel.

Previously we observed that one RBC at the bifurcation causes an increase in pressure drop, whereas one RBC is far from the physiological situation. Therefore, in this study, we quantified the change in pressure drop when multiple RBCs move in the vessel. Figure 11 shows the distribution of the pressure drop in the vessel during RBC motion. The pressure drop in the parent was essentially the same for all the groups. When RBCs flowed into the bifurcation, the pressure drop increased sharply and then decreased sharply at the bifurcation. The largest pressure drop for D-RBCs is 167.67 Pa at the bifurcation, while that for H-RBCs is 155.82 Pa, implying that in a specific vessel, D-RBC causes a higher flow resistance compared to H-RBC.

## 4. Discussion

First, we analyzed the effects of λ and υ in shear flow on the dynamic behavior of RBC. When $\lambda ≺\lambda_{c}$, RBCs perform TT motion, and when $\lambda \geq \lambda_{c}$, RBCs mainly perform TB motion. We found that the TT motion and TB motion speed of the D-RBC are greater than those of the H-RBC, which is consistent with the results of previous experiments [43]. The TT motion perimeter becomes longer as the RBC is stretched in shear flow, while the biconcave shape and diameter of the D-RBC also decreased to some extent, which led to a slightly greater TT frequency in the D-RBC than in the other two groups. In addition, the slowing down of the TT motion with an increasing viscosity ratio is due to the increased viscosity in the cytoplasm, which slows down the rotation of the cytoplasm and motion of the RBC membrane [44].

As the cytoplasmic viscosity increases further, a shift from the TT motion to TB motion can be induced. This is due to the fact that as the viscosity increases, the transfer of shear torque to the membrane becomes increasingly difficult, at which point the RBC behaves like a near rigid solid and shows TB motion [45]. The larger the λ, the faster the TB frequency. It is closer to a rigid particle, which leads to a higher TB frequency as the viscosity of the RBC increases. It is possible that increased viscosity makes it less likely to flow, and a decrease in the RBC deformability leads to a significant increase in the TB motion [46,47].

Second, we analyzed the velocity, pressure drop, and lingering time of the RBC in the retinal bifurcating vessel. Our simulations revealed that the transit time of the H-RBC throughout the retinal bifurcating vessel was lesser than that of the D-RBC and P-RBC. This difference mainly occurs near the bifurcation and downstream of the bifurcation. Peduzzi et al. [48] showed that blood rheology disorders can cause capillary and post-capillary small venous stagnation in patients with DM and that stagnation is a major cause of blindness in patients with DM. The increased lingering time of RBC at the bifurcation decreases the blood flow in the retinal vessels, and the stagnation of microcirculatory blood flow leads to local hypoxia and lactic acidosis, which in turn causes microvascular damage [49]. Related studies have shown that a concomitant increase in blood viscosity caused by hyperglycemia [50] and increased RBC viscosity in DM leads to decreased RBC deformability and mobility, which increases the intrinsic resistance to blood flow in the vasculature [51,52]. The transit time of RBC increases with increased blood glucose [53], indicating that the retinal vasculature is susceptible to hyperglycemic injury, and the presence of hyperglycemia has given importance by patients.

In addition, we found that the local pressure drop at the bifurcation increased and the overall pressure drop in the D-RBC and P-RBC flowing in the vessel was greater than that in the H-RBC. This is due to the lingering of RBC at the bifurcation, which leads to a decrease in flow into the child vessel and an increased pressure near the entrance of the child vessel. Therefore, a sharp rise in the pressure drop near the bifurcation, leads to a significant increase in the resistance to flow, which is consistent with the findings of Peter Balogh [13]. In the DM state, increased glycosylation, cholesterol, and oxidative stress may lead to swelling of RBCs with impaired deformability [54]. Thus, the combination of the reduced biconcave shape of the D-RBC and physical parameters leads to a higher local pressure drop than that in the H-RBC. Several qualitative studies have been performed to study the effects of RBC physical parameters on hemodynamics. We additionally investigated the effect of RBC shape on hemodynamics (the results are not yet published). Keeping other parameters constant, the reduced biconcave shape of RBC still increases the transit time and flow resistance, indicating that the hemodynamic parameters are highly sensitive to RBC morphology and that the alteration of RBC morphology is a potential marker of microvascular disease.

Finally, we analyzed the velocity and pressure drop of multiple RBCs in a bifurcating retinal vessel. The pressure drop induced by D-RBCs at the bifurcation is significantly greater than that induced by H-RBCs, indicating that D-RBCs induce greater resistance to the flow in retinal vessels, which may reflect more pronounced hemodynamic disturbances in retinal vessels in DM. The enhanced aggregation of D-RBCs and P-RBCs at the bifurcation, increased lingering time, and decreased RBC deformability resulted in a dramatic increase in local pressure drop, and created greater flow resistance, which is the result of a combination of reduced RBC biconcave shape, increased viscosity, and RBC membrane stiffness. This suggests that the DM state is more prone to vascular occlusion, leading to local hypoxia. Kiani et al. [55] simulated flowing deformable disc-shaped particles and found an order-of-magnitude increase in the pressure gradient at a bifurcation. P-RBCs also caused a certain degree of rising pressure drop relative to H-RBCs. Previous studies have shown that a hyperglycemic state can induce the accumulation of oxidative stress, which promotes cellular damage and the development of DM complications [56,57]. As the blood glucose level increases, RBC aggregation is also enhanced [53]. Vekasi et al. [58] noted that chronic hyperglycemia may lead to disturbances in blood rheology, leading to further microcirculatory disturbances. D-RBCs exhibit an increased adhesion to the vascular endothelium at bifurcation [59], and RBCs and endothelial cells may act synergistically to severely impede retinal perfusion and trigger retinopathy. Previous studies have shown that a significantly higher number of endothelial cells at bifurcation occurs in patients with DM than in other patients [60]. This is due to the altered structure and function of RBCs, which can increase their aggregation and adhesion to endothelial cells [61]. Therefore, a decrease in the biconcave shape of RBC and an increase in the viscosity and RBC membrane stiffness may exacerbate the process of disease.

In this study, although the thickness of the RBC membrane was taken into account, the effect of the actual composition of the RBC membrane on its movement was not thoroughly considered. In addition, due to the limitation of computing resources, the calculated retinal blood vessel is a symmetric idealized model, and the real microvessel network with the development of the disease has not been established. In the 3D modeling work of biomechanics of D-RBC by Chang et al. using spring network model [35], the computed frequency of the angular trajectory for the marked particle of the cell membrane is larger than ours, implying that the overall two-dimensional model can solely represent part of the RBC behaviors in microvessel network. In the future, a three-dimensional RBC continuum model should be developed in the next work. A foreseeable extension of the work would be to investigate the RBC movement and vasodilation function in the real retinal vascular network model extracted from available imaging data, so that more clinical supports can be used for comparison.

## 5. Conclusions

First, hemodynamic calculations were performed for H-RBCs, P-RBCs, and D-RBCs in shear flow, and the effects of RBC morphology and physical parameters on RBC dynamic behavior were investigated. The TT motion, TB motion, and linear relationship between TT frequency, TB frequency, and shear rate are consistent with the results of previous studies, indicating that the finite element method can successfully predict the complex dynamics of RBC in the shear flow under different periods. We found that abnormal variations in the morphological and physical parameters of RBCs may lead to diminished tank-tread motion and enhanced tumbling motion in shear flow.

Second, single and multiple RBC motions were simulated in the retinal bifurcation vessels to compare changes in hemodynamic parameters of H-RBCs, P-RBCs, and D-RBCs in the retinal bifurcation vessels. The results show that as DM progresses, the RBC lingering time becomes longer at the bifurcation, resulting in sharply increasing local pressure and a greater resistance to flow, leading to longer transit times of RBCs throughout the vessel, and which may promote further development of DM, and provoke microcirculatory complications.

## Figures and Tables

**Table 1:** Values for parameters used in the model.

Symbol	Value, Units	Description
$d_{0}$	9.5 μm	parent vessel diameter
k	2.36	vessel index
ψ	1	asymmetry index
$\rho_{fluid}$	1060 kg/m^3^	plasma density and cytoplasmic density
$\rho_{solid}$	1090 kg/m^3^	RBC membrane density
$\lambda_{c}$	5	physiological viscosity ratio
λ	1, 3, 5, 7, 9	viscosity ratio
υ	50, 100, 150 1/s	shear flow rate

**Table 2:** Parameters used in the numerical simulation.

Parameters	H-RBC	P-RBC	D-RBC
Diameter (μm)	6.91 [26]	6.82 [26]	6.89 [26]
Thickness (μm)	2.36 [26]	2.4 [26]	2.68 [26]
Concave depth (μm)	0.43 [26]	0.37 [26]	0.143 [26]
$\mu_{plasma}$ (Pa·s)	0.00128 [32]	0.00131	0.00133 [32]
E (kPa)	1820 [8]	2020	2520 [8]
τ (s)	0.1 [33,34]	0.08 [35]	0.07 [35]

## Data Availability

Correspondence and requests for materials should be addressed to L.L.
